# Human Urinary Kallidinogenase Promotes Angiogenesis and Cerebral Perfusion in Experimental Stroke

**DOI:** 10.1371/journal.pone.0134543

**Published:** 2015-07-29

**Authors:** Lijuan Han, Jie Li, Yanting Chen, Meijuan Zhang, Lai Qian, Yan Chen, Zhengzheng Wu, Yun Xu, Jingwei Li

**Affiliations:** 1 Departments of Neurology, Drum Tower Hospital, Medical School of Nanjing University, Nanjing, China; 2 The State Key Laboratory of Pharmaceutical Biotechnology, Nanjing University, Nanjing, China; 3 Jiangsu Key Laboratory for Molecular Medicine, Medical School of Nanjing University, Nanjing, China; 4 Jiangsu Province Stroke Center for Diagnosis and Therapy, Nanjing, China; 5 Nanjing Neuropsychiatry Clinic Medical Center, Nanjing, China; 6 Department of Neurology, Affiliated Yixing People's Hospital of Jiangsu University, Yixing, China; School of Pharmacy, Texas Tech University HSC, UNITED STATES

## Abstract

Angiogenesisis a key restorative mechanism in response to ischemia, and pro-angiogenic therapy could be beneficial in stroke. Accumulating experimental and clinical evidence suggest that human urinary kallidinogenase (HUK) improves stroke outcome, but the underlying mechanisms are not clear. The aim of current study was to verify roles of HUK in post-ischemic angiogenesis and identify relevant mediators. In rat middle cerebral artery occlusion (MCAO) model, we confirmed that HUK treatment could improve stroke outcome, indicated by reduced infarct size and improved neurological function. Notably, the ^18^F-FDG micro-PET scan indicated that HUK enhanced cerebral perfusion in rats after MCAO treatment. In addition, HUK promotespost-ischemic angiogenesis, with increased vessel density as well as up-regulated VEGF andapelin/APJ expression in HUK-treated MCAO mice. In endothelial cell cultures, induction of VEGF and apelin/APJ expression, and ERK1/2 phosphorylation by HUK was further confirmed. These changes were abrogated by U0126, a selective ERK1/2 inhibitor. Moreover, F13A, a competitive antagonist of APJ receptor, significantly suppressed HUK-induced VEGF expression. Furthermore, angiogenic functions of HUK were inhibited in the presence of selective bradykinin B1 or B2 receptor antagonist both *in vitro* and *in vivo*. Our findings indicate that HUK treatment promotes post-ischemic angiogenesis and cerebral perfusion via activation of bradykinin B1 and B2 receptors, which is potentially due to enhancement expression of VEGF and apelin/APJ in ERK1/2 dependent way.

## Introduction

Ischemic stroke is caused by critical reductions in blood flow to one or more arteries of the brain or spinal cord, and is a leading cause of morbidity and mortality worldwide. However, the only clinically- validated treatment for stroke now available is acute thrombolysis tissue-type plasminogen activator (tPA), and the utilization of this approach is constrained by the need to initiate treatment rapidly after the stroke onset and the risk of causing cerebral hemorrhage [1]. It is therefore important to develop new approaches that can be used for a much larger fraction of stroke patients. For several decades, most attempts have been focused on neuroprotection that refers to single drug therapy or combinations therapy to antagonize the injurious biochemical and molecular events contributing to irreversible ischemic injury [2, 3]. Recently, more and more attention has been drawn to neurorestorative therapies, which possess far longer time window than acute neuroprotection [4, 5]. Angiogenesis, a key neurorestorative event, primarily occurs in the ischemic boundary zone. The generation of new blood vessels facilitates highly coupled neurorestorative processes including neurogenesis and synaptogenesis, which result in improved functional recovery [6–8].

Tissue kallikrein, an important component of the kallikrein/kinin system (KKS), is a serine proteinase capable of cleaving low molecular weight kininogen to release vasoactive kinins, which in turn activate bradykinin B1 and B2 receptors (B1R and B2R) and trigger a series of biological effects [9]. Accumulating evidence suggest that tissue kallikrein therapy is a promising regimen in the treatment of acute ischemic stroke [10]. Compared with tPA, tissue kallikrein has superior advantage with a wider time window of several days [11]. In addition, Kallikrein has been identified to protect against ischemic brain injury through multiple mechanisms including anti-inflammation, anti-apoptosis, promoting angiogenesis and neurogenesis [12–14]. The angiogenic roles of kallikrein were also demonstrated in the ischemic research including hindlimb ischemia, myocardial infarction and renal ischemia [15–17].

Human urinary kallidinogenase (HUK) is a tissue kallikrein extracted from urine. HUK has been approved by China’s State Food and Drug Administration as a state category I new drug for the treatment of stroke patients. Based on the available evidence, HUK injection ameliorates neurological deficits and improves long-term outcomes [10]. Previous animal studies have indicated that HUK could inhibit the decrease of cerebral blood flow and improve cerebral glucose metabolism, and suppress brain edema and block post-stroke inflammatory cascades [13]. In this study, we evaluated angiogenic roles of HUK in experimental stroke and the underlying molecular mechanisms.

## Materials and Methods

### Ethics statement

The animal study protocols were approved by the Committee of Experimental Animal Administration in Nanjing University. The study was conducted on male SD rats (body weight 250–300 g), which were provided by the Animal Center of Drum Tower Hospital, and were housed in specific pathogen free (SPF) laboratoryin the course of theexperiment. The SD rats were sacrificed by pentobarbital sodium(over 100 mg/kg, intraperitoneally). All efforts were made to minimizeanimalsuffering during the study.

### Rat middle cerebral artery occlusion (MCAO) model and drug treatment

MCAO model was performed as previously described with some minor modifications [18]. In brief, a 4–0 monofilament nylon suture with heat-rounded tip was inserted from the external carotid artery into the origin of the middle cerebral artery. After 2 h of ischemia, blood reperfusion was initiated by filament withdrawal. Heart rate and blood pressure were monitored from pre-MCAO to 1.5 h after the HUK treatment using the tail-cuff method (PowerLabo/S system connected to CHART software; AD Instruments, Milford, MA, USA). The femoral artery of rat was cannulated to detect the arterial blood. Femoral artery blood (0.5mL) was obtained for blood gas using a clinical blood gas analyzer (Radiometer, Copenhagen, Denmark). Sham- operated rats were subjected to the same procedure without MCAO. HUK (Techpool Bio-Pharma Co. LTD, Guangdong, China), with molecular weight of 43±4 KD, purity greater than 98% and endotoxin level less than 25 EU/ PNA U, was dissolved with saline and prepared in the clinic way. HUK-treated rats received an injection of HUK through vena caudalis immediately after reperfusion at the dosage of 1.6 × 10^−2^ PNA U/kgover a period of 5 minutes and 0.9% saline solution as a vehicle as described previously [19]. Selective bradykinin B1 receptor antagonist (R715, 0.5mg/kg) and selective B2 receptor antagonist (HOE140, 100μg/kg) was administrated 30 minutes prior to HUK injection to block the activity of B1 and B2 receptor in stroke rats, respectively. Rats in each group were sacrificed at 3 h, 1 d, 3 d, 7d, and 14 d after MCAO for the following studies.

### Determination of infarct size

5-triphenyltetrazolium (TTC) Staining was used to determine the infarct size of MCAO rats. Six rats of each group were sacrificed 1, 3, 7 d after reperfusion and were sliced into 2 mm thick coronal sections and then immersed in a 2% TTC (Sigma, St Louis, MO, USA) solution at 37°C for 30minutes. Slice staining was photographed with a computer-controlled digital camera (Olympus, Japan). Infarct volume was calculated using Image-Pro Plus 6.0 (IPP) software (Media Cybernetics). The value of the infarct volume was presented as the percent of the contralateral hemisphere after a correction for edema in the ipsilateral cortex.

### Assessment of neurological function

Neurologic evaluation was performed at 3 h, 1 d, 3 d, 7 d and 14 d after the onset of occlusion until sacrifice. Rats were sacrificed after neurologic evaluation at indicated time points. According to Longa Score [20], neurological function was modified and graded on a scale of 0 to 4: a score of 0 indicated no neurologic deficit, rats with a score of 1 failed to extend left forepaw fully a mild focal neurologic deficit, a score of 2 (circled to the left) a moderate focal neurologic deficit, and a score of 3 (fell to the left) a severe focal deficit; rats with a score of 4 did not walk spontaneously and had a depressed level of consciousness.

### Micro-PET

Micro-PET images of the rat brain were acquired immediately after rat MCAO surgery and 1 d and 14 d after drug treatments. Before the scanning, all rats were deprived of food for 8–12 h to enhance FDG uptake in the brain. Under anesthesia using isoflurane (1.5%) FDG (1.0–1.5mCi/kg) was administered via tail vein injection for all rats. Micro-PET scanning information was recorded, including weight, injection time, dose, residual dose measurement, measuring residual time and so on. After 60 min of FDG uptake, the anesthetized rats were fixed on the scan bed for 10-minute Micro PET scan. Image acquisition was performed using a special small-animal PET scanner (Inveon, Germany SIMENS company), dealing with the OSEM3D format to obtain the outline of region of interest (ROI).

### RNA isolation and real-time PCR

Briefly, total RNA was extracted with Trizol reagent (Invitrogen, Carlsbad, USA) and reversed to cDNA using a PrimeScript RT reagent kit (Takara, Dalian, China) according to the manufacturer's instructions. Quantitative PCR was performed in the presence of a fluorescent dye (Takara, Dalian, China) by ABI 7500 machine (USA). Quantity of mRNA was analyzed after normalization to glyceraldehyde-3-phosphate dehydrogenase (GAPDH) ribosomal RNA.

Primer sequences are as follow:

Apelin: F: GCTCGCGCCTTTCTTTGCGT,

R: TCACCAGGTAGCGCATGTTGCC;

APJ: F: TGCCCGTCATGGTGTTCCGT,

R: AGGGCACCACAAAGCCCACA;

CD31: F: TTTCGCTGCCAAGCTGGCGT,

R: CCACCTGCACGCTGCACTTGAT;

VEGF: F: AAGTGACCACATTCACTGTGAGCCT,

R: GCTCACCGCCTTGGCTTGTCA;

GAPDH: F: GGCTCTCTGCTCCTCCCTGTTCTA,

R: CGGCCAAATCCGTTCACACCGA.

### Western blotting

Proteins from brain tissue were extracted and quantified by Bio-rad protein extraction kit (Bio-Rad, Hercules, CA, USA) according to the manufacturer’s instructions. Equal amounts of total protein samples were separated by sodium dodecyl sulfate-PAGE and blotted onto polyvinylidene fluoride membranes. The membranes were probed with primary antibodies against VEGF (1:1000, Abcam, USA), GAPDH (1:2,000, Bioworld, USA), ERK1/2 (1:1,000; Cell Signaling, USA). Horseradish peroxidase-conjugated anti-rabbit or anti-mouse secondary antibodies were then used and the reaction was observed using chemiluminescence reagents provided with the ECL kit (Bio-Rad, Hercules, CA, USA) and exposed to a film. The intensity of blots was quantified by densitometry.

### Immunostaining

At the designated survival intervals, the rats were anesthetized and transcardially perfused with 0.9% saline and followed by 4% paraformaldehyde (PFA). Twenty- brain slices were incubated with anti-Rat CD31 primary antibody (1:50, Santa Cruz, CA, USA) overnight at 4°C and then secondary antibody Alexa Fluor 594 anti—rabbit IgG (1:200, Invitrogen, USA) for 2 h at room temperature. Cell nuclei were stained with DAPI. Stained samples were imaged using an Olympus microscope with a BX51 digital camera. Fluorescent images were processed using IPP6.0 software. Quantification analysis was performed on 3 sections per hemisphere. In each brain, three to four randomly fields of ischemia cortex were selected on each section.

### Endothelial cell culture

Human artery endothelial cells (HAECs) were purchased from Cambrex Bio Science (San Francisco, CA, USA) and were used between the third and sixth passages. The cells were cultured in endothelial basal medium (EBM)-2 containing EGM-2-MV SingleQuots (BioWhittaker). HUK was added to the medium with a final concentration of 0.1 μM. U0126 (10 μM), a selective inhibitor of ERK1/2, was added to the medium 30 minutes before HUK treatment. HAECs incubated with HUK alone or HUK plus U0126 for 15 min, 1 h, 3 h and 24 h were used for Q-PCR and Western blotting experiments.

### Tube formation assay

HAECs cultured in 96-well plates were used to perform the tube formation assay as previously described. Corning Matrigel Basement Membrane Matrix Growth Factor Reduced (BD Biosciences, Bedford, MA, USA) was thawed on ice under 4°C over night. 60 μlMatrigelper well was added to pre-chilled 96-well plate and was allowed to be solidified for 1 hour under 37°C. Then, endothelial cells were plated at a density of 2 × 10^4^ cells/well. HAECs were exposed to oxygen-glucose deprivation (OGD), an *in vitro*model simulating ischemia, for 3 hours followed by the treatment of HUK in the presence of R715(0.5 μM) or HOE140(1μM). Formation of tube structure was evaluated 6 hours later. Images were acquired using Leica DFC345 FX microscope. Total tube length of capillary-like structure was measured usingImageJ software in three randomly selected fields (10×) for each group. Data was presented as total tube length/mm^2^ from three independent experiments.

### Statistical analysis

All data are presented as means±standard error of the mean (SEM) and were analyzed by the SPSS 19.0 statistical analytical software (SPSS, Chicago, IL, USA). Differences between multiple groups were analyzed by the one-way analysis of variance (ANOVA) method. Change between two groups was statistically evaluated by Student’s t-test. Comparative differences were considered significant at *P*<0.05.

## Results

### HUK protects against ischemic brain injuries in rats

**Fig 1:**
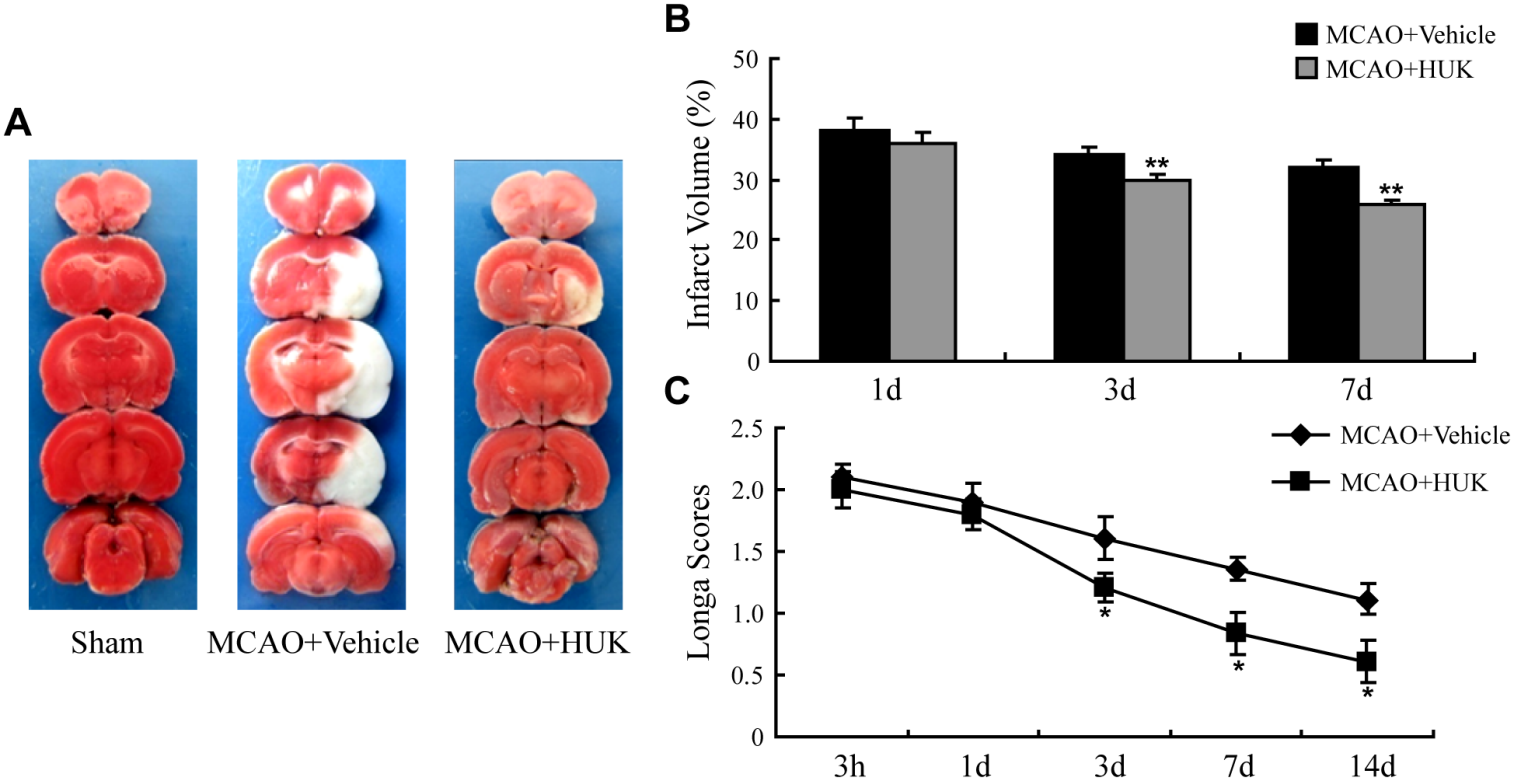
HUK protected against ischemic brain injury. (A) Representative TTC images showing infarct size 3 d after MCAO. The normal tissue was stained deep red and the infarct area was stained pale gray. (B) Statistic bar graph of brain infarct size in vehicle or HUK-treated rats at 1 d, 3 d, and 7 d after MCAO. **p<0.01 *versus* vehicle group, N = 6. (C) Neurological performance of rats in each group assessed with longa score at 3 h, 1 d, 3 d, 7 d, and 14 d after reperfusion. *p<0.05 *versus* control group, N = 10 per group.

To investigate effects of HUK on stroke outcomes, infarct volume and neurological deficit were evaluated in MCAO rats with vehicle or HUK treatment. It was found that HUK apparently reduced the infarct volume of MCAO rats at 3 d and 7 d after reperfusion (Fig 1A and 1B). The difference of neurological function between MCAO rats with or without HUK treatment was also compared. As shown in Fig 1C, rats subjected to MCAO demonstrated neurological deficit, which was alleviated by HUK treatment at 3 d, 7 d and 14 d time points after cerebral ischemia (Fig 1C). In addition, major physiological parameters including blood gas, heart rate and blood pressure were unaffected by HUK treatment in MCAO rats (S1A–S1C Fig).

### HUK enhanced cerebral blood perfusion in stroke rats

**Fig 2:**
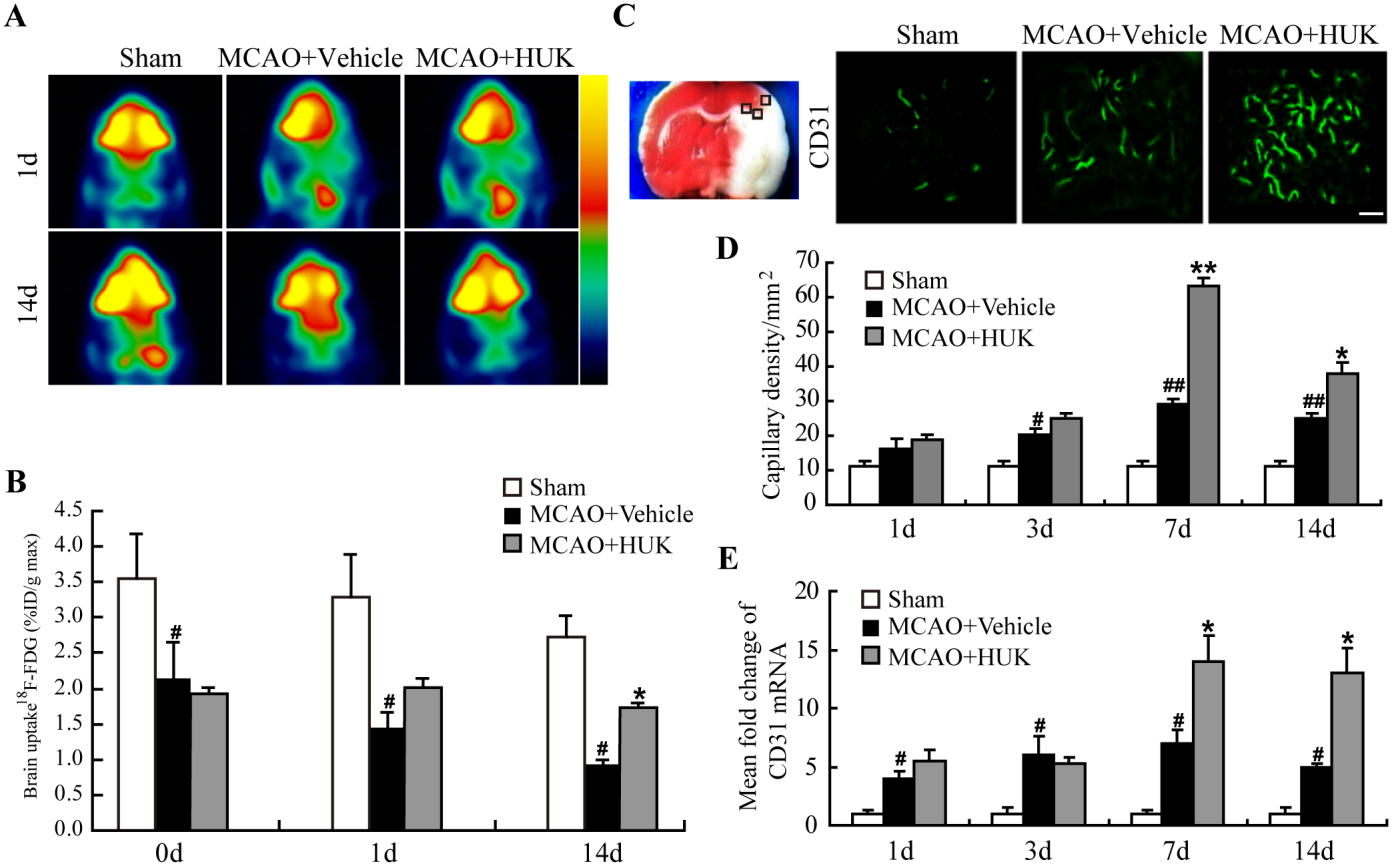
HUK enhanced cerebral blood perfusion and promoted angiogenesis in stroke rats. (A) Representative images of ^18^F-FDG PET/CT in HUK group and vehicle group at 1 d and 14 d after stroke. (B) Uptake of ^18^F-FDG in HUK or vehicle-treated rats at different time points after stroke. *p<0.05 *versus* vehicle group; **p<0.01 *versus* vehicle group, N = 10 per group. (C) Left panel showed the typical detected areas of CD31 staining in rats brain indicated in boxed area. Right panel demonstrated representativeimmunofluorescent images stained by CD31 (green) 7 days after reperfusion. Scale bar = 100 μm. (D) CD31 positive vessels were counted and analyzed at indicated time point. Data was presented as capillary density reflected by number of CD31 positive vessels per mm^2^. #*p*<0.05 *versus* sham group; ##*p*<0.01 *versus* sham group; *p<0.05 *versus* vehicle group; **p<0.01 *versus* vehicle group. N = 6 per group. (E) Quantitative data of CD31 mRNA levels at 1, 3, 7, and 14 d after stroke onset. # *p*<0.05 *versus* sham group; *p<0.05 *versus* control group. N = 6 per group.

To determine whether HUK could improve cerebral blood perfusion of ischemic brain, 18F-FDG PET/CT scan was performed in rats up to 14 d after MCAO. It was found that acute cerebral ischemia induced a profound decrease in the uptake of 18F-FDG in rats (Fig 2A and 2B). As expected, the uptake of 18F-FDG of ischemic hemisphere in HUK group was significantly higher than that in vehicle group at 14 d after stroke (Fig 2A and 2B).

### HUK increased angiogenesis in stroke rats

Brain collateral circulation contributes to cerebral blood perfusion after stroke if no recanalization of the vessel with thrombosis. We focused on the third level collateral circulation- angiogenesis, which was evaluated by measurement of microvessel density indicated by endothelial cell marker CD31 in the peri-infarct cortex of stroke rats (Fig 2C). It was observed that CD31 expression increased as early as 1 d after cerebral ischemia, suggesting angiogenesis occurred early after stroke onset (Fig 2C and 2D). Compared to vehicle-treated MCAO rats, capillary density reflected by CD31-positive stainingper area was significantly up- regulated at 7 d and 14 d in the cortex of HUK- treated MCAO rats (Fig 2C and 2D). In addition, mRNA expression of CD31 demonstrated similar change pattern after treatments (Fig 2E).

**Fig 3:**
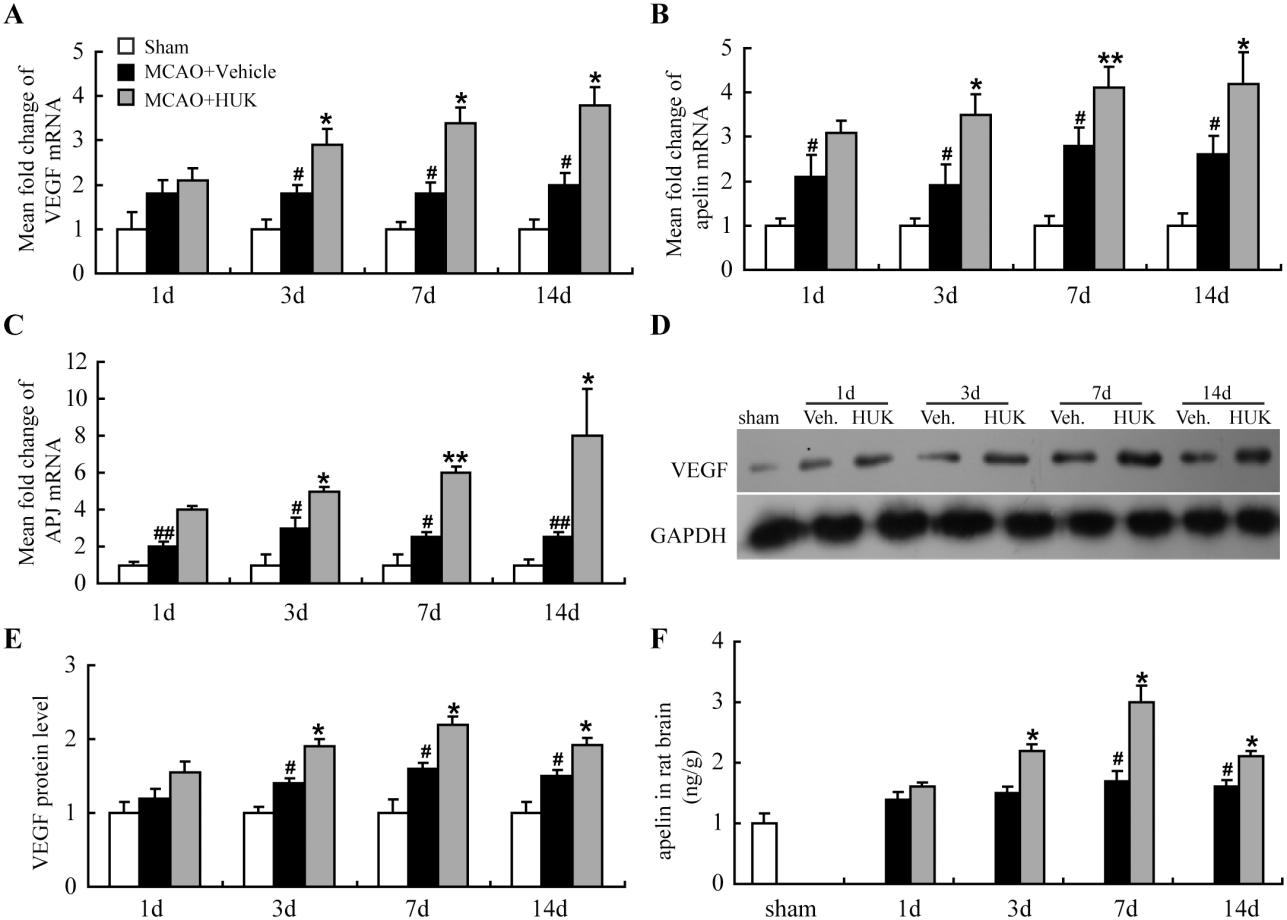
HUK enhanced VEGF, Apelin/APJ expression in stroke rat brain. MCAO rats with or without HUK treatment were sacrificed and mRNA was extracted for the detection of apelin (A), APJ (B), VEGF (C) levels by real-time PCR. N = 6 per group. #*p*<0.05 *versus* sham group; ##*p*<0.01 *versus* sham group; *p<0.05 *versus* control group; **p<0.01 *versus* control group. Protein level of apelin in rat brain was determined by ELISA (F), while protein level of VEGF in rat brain was measured by Western blot (D). Quantitative data of VEGF blots at indicated time points (F). #*p*<0.05 *versus* sham group; *p<0.05 *versus* vehicle group, N = 6 repeats.

VEGF is a well-known growth factor regulating vascular formation and maturation [8]. In addition, apelin is a recently indentifiedangiogenic factor, acting by interaction with its specific receptor APJ, under physiological and pathological conditions [21]. Several studies demonstrated that apelin and APJ receptor was present in human and rat endothelial cells, including endothelial cells from rat small cerebral vessels.[22–24] Furthermore, analysis of double immunostaining revealed a similar intra-cellular localization pattern of apelin and APJ receptor in cultured HUVECs,[24] indicating an important role of apelin/APJ as a potential vasculature regulatory signaling. With the aims to find out the molecular mechanisms through which HUK promoted angiogenesis, the mRNA expression of angiogenic factors including VEGF and apelin/APJ in the rat brain tissue was examined at indicated time points after MCAO. Our data revealed that VEGF, apelin and APJ transcripts were up-regulated at 3, 7 and 14 d after HUK injection (Fig 3A–3C). In addition, the alternation of VEGF protein level was also verified by western blotting as shown in Fig 3D and 3E (HUK group *vs*. Vehicle group: 1.28 folds at 3 d, *p*<0.05; 1.30 folds at 7 d, *p*<0.05; 1.26 folds at 14 d, *p*<0.05). In consistent with apelin mRNA level, Apelin protein expression was also increased by HUK,with peak expression at 7 d (Fig 3F). Thesefindings indicated that enhanced VEGF and Apelin/APJ might play roles inangiogenic effects of HUK in ischemic stroke.

### HUK modulated pro-angiogenic factors expression in ERK1/2-dependent way in cultured endothelial cells

**Fig 4:**
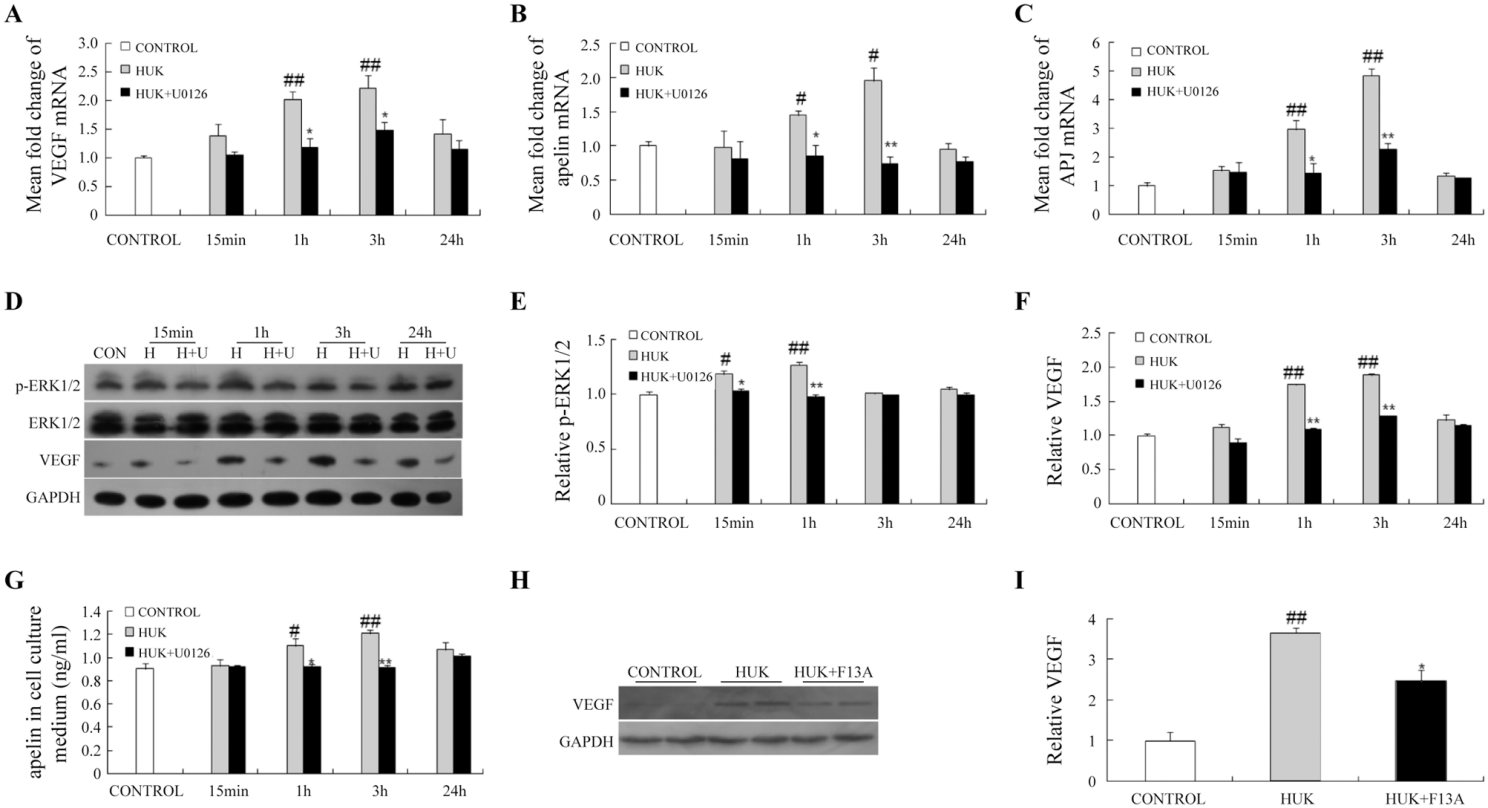
HUK up-regulated pro-angiogeic factors in ERK1/2 dependent way in cultured endothelial cells. mRNA from cultured HAECs treated with HUK only or HUK plus U0126 was isolated for the measurement of VEGF (A), apelin (B), and APJ (C). N = 6 per group. (D) Western blot showed the activation of ERK pathway and VEGF expression in cultured HAECs incubated with HUK only or U0126 plus HUK for 15 min, 1 h, 3 h and 24 h. Statistic bar graph of p-ERK/ERK blots (E) and VEGF blots (F), N = 6 per group. (G) ELISA was used to measure apelin concentration in cultured cell medium. (H) F13A decreased VEGF expression up- regulated by HUK. (I) Quantitative data of VEGF blots intensity with or without F13A. #*p*<0.05 versus control group *p<0.05 versus HUK group; **p<0.01 versus HUK group; ##*p*<0.01 versus control group. N = 6 per group.

Human artery endothelial cells (HAECs) cultures were then isolated and cultured to further investigate the underlying mechanisms of angiogenic functions induced by HUK *in vitro*. Consistent with the data *in vivo*, HUK significantly augmented pro-angiogenic factors, including VEGF, apelin and APJ levels in cultured HAECs determined by real-time quantitative PCR (Fig 4A–4C). In cultured medium, HUK increased apelin at 1 h and 3 h time points after its incubation with cells measured by ELISA (Fig 4G).

**Fig 5:**
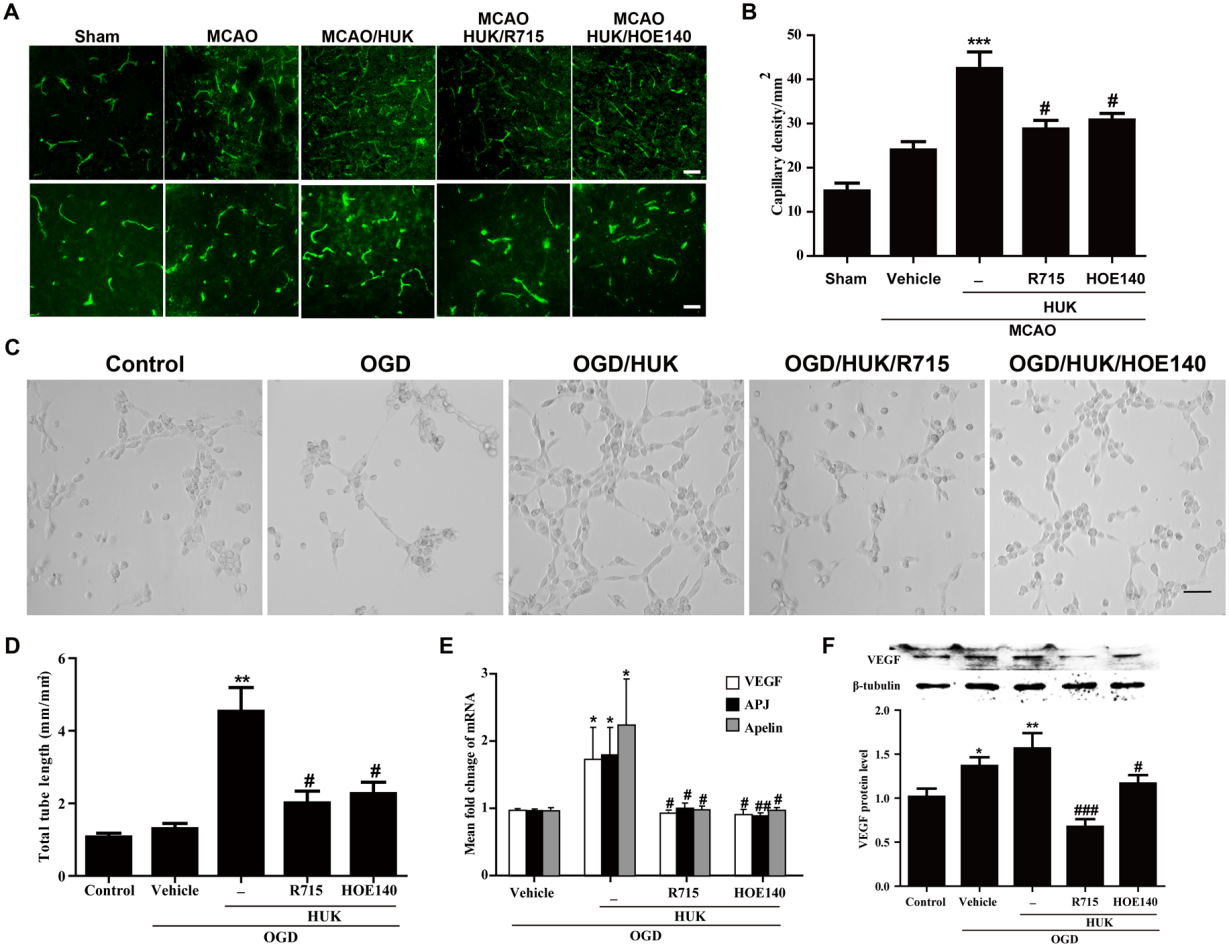
HUK promoted angiogenesis through activation of bradykinin B1 and B2 receptors. (A) Representative images of CD31 immunostaining (green) in rats peri-infarct cortex 7 days after reperfusion. Both R715 (0.5mg/kg) and HOE140 (100μg/kg) reduced capillary density induced by HUK in stroke rats. Scale bar in upper panel = 100 μm, in lower panel = 50 μm. (B) Capillary density was quantified by CD31 positive vessels per mm^2^. (C) Representative images of tube formation assay. 30 minutes pre-treatment with R715 (0.5 μM) orHOE140 (1 μM) inhibited HUK-induced increase in tube formation capacity of OGD-treated HAECs. Scale bar = 100 μm (D) Total tube length of capillary like structure was analyzed and presented as total tube length permm2. (E) Real-time PCR demonstrated that blockade of either B1 or B2 receptors inhibited HUK-induced increase in mRNA levels of VEGF, apelin, and APJ of OGD-treated HAECs. (F) Upper panel showed VEGF protein expression detected by western blot. Lower panel demonstrated quantification of VEGF blots intensity using Image J software. Protein expression of VEGF was normalized to internal control, β-tubulin. Blockade of either B1 or B2 receptors inhibited HUK-induced VEGF expression at protein levels of OGD-treated HAECs. *** p<0.001 versus Sham group, **p<0.01 versus control group, *p<0.05 versus vehicle group; #p<0.05 versus HUK group. N = 6 per group.

Our previous study reported the HUK increased ERK1/2 phosphorylation in experimental ischemic mouse brain. In this study, we incubated cultured endothelial cells with HUK and found that phosphorylated ERK1/2 began to increase at 15 min, reached a high level at 1 h, and then gradually returned to the baseline level (Fig 5E). Inhibition of ERK1/2 activation by U0126 (10μM) impeded ERK1/2 phosphorylation, and resulted in blockade of APJ, VEGF and apelin expression induced by HUK (Fig 4A–4G). These data suggested that HUK-triggered up-regulation of VEGF, apelinandAPJ in HAECs was dependent on the ERK1/2 activation.

F13A is a competitive antagonist blocking APJ receptor with a substitution of phenylalanine by alanine at the C-terminal of apelin-13. In order to further elucidate the crosstalk between apeligenic system and VEGF, we pretreated HAECs with F13A (2nM) and subsequently HUK (0.1 μM) for 12 hours. As shown in Fig 4H and 4I, VEGF expression partially decreased accompanied with the blockade of apelin/APJ system, indicating that VEGF subjected to the regulation of apelin in HAECs and apelin may work cooperatively with VEGF in stimulating vessel growth.

### HUK promoted angiogenesis through activation of bradykinin B1 and B2 receptors

Kininsgenerated from kininogenthrough cleavage by kallikreins along with its metabolites, have been demonstrated to regulate various biological effects, including angiogenesis, via binding tobradykinin B1 and B2 receptors [9]. To investigate whether HUK promoted angiogenesis via bradykinin receptors, selective B1 receptor antagonist R715 and selective B2 receptor antagonist HOE140 was used to block B1 and B2 receptor in stroke rats, respectively. As shown in Fig 5A and 5B, HUK-induced increase in capillary density in stroke rats at 7 d after MCAO was significantly blocked by both R715andHOE140 pre-treatment. Meanwhile, we further tested the involvement of bradykinin receptors in the angiogenic effect of HUK *in vitro* using a tube formation assay, an *in vitro*experiment to assess the angiogenicpotential by measuring tube formation capacity of endothelial cells. HAECs were exposed to OGDfor 3 hours, and then treated with HUK in the presence or absence of 30 minutes pre-treatment with R715 (0.5 μM) orHOE140 (1 μM). Consistent with the findings *in vivo*, capillary like structure was irregular in the presence of R715orHOE140compared to HUK alone treatment (Fig 5C). Quantification of total tube length showed thatR715 and HOE140pre-treatment significantly reduced the tube formation capabilityofHUK in OGD-treated HAECs (Fig 5D). Additionally, HUK-induced increase in the expression of VEGF, apelin and APJ at mRNA level was decreased by both R715 and HOE140 pre-treatment in HAECs 3 hours after OGD exposure (Fig 5E). Meanwhile, results of western blot demonstrated that blockade of either B1 or B2 receptor also prevented HUK-induced VEGF protein expression in OGD-treated HAECs (Fig 5F). Taken together, these data indicated that activation of either B1 or B2 receptors were involved in the angiogenic effect of HUK in stroke rats and endothelial cells.

## Discussion

In this study, we first confirmed neuroprotective effects of HUK (exogenous human tissue kallikrein) in rat stroke model. Then we for the first time identified: 1) HUK enhanced cerebral blood perfusion and facilitated angiogenesis in rats after stroke; 2) HUK promoted VEGF andapelin/APJ expression in MCAO rats and endothelial cell cultures; 3) The underlying molecular mechanism potentially involved withenhancement expression ofVEGF and apelin/APJ in ERK1/2 dependent way;4) angiogenic effect of HUK was associated with activation of B1 and B2 receptors.

The kallikrein—kinin system (KKS)is composed of kinins, kallikreins and kininogens. It is widely accepted thatthe KKS play a role under normal physiological conditions, anddisturbances of the KKS may account for the pathogenesis of many diseases including cardiovascular disorders, cerebrovascular disease and renal diseases [9, 11, 25, 26]. Recent evidence suggests that the tissue kallikreinKKS, one key component of the KKS, is implicated in multiple pathological stages and represents an attractive therapeutic target in acute ischemic stroke [11, 12, 27, 28]. Previous studies have demonstrated that delivery of human tissue kallikrein gene, protein or peptide protects against cerebral ischemia/reperfusion (I/R) injury through inhibition of oxidative stress and apoptosis, enhancement of glial cell survival, and migration and promotion of angiogenesis and neurogenesis [29, 30]. In this study, injection of HUK that supply human tissue kallikrein could reduce infarct size and improve neurological deficit in stroke rats (Fig 1), consistent with the findings of another animal study from our lab [13]. In addition, a recent clinical systemic review on the efficacy and safety of HUK in stroke studied 2433 patients (24 trials) in China and demonstrated that 2117 patients (22 trials) benefited from HUK treatment, eliciting 87% efficacy rate [10].

Tissue kallikrein gene transfer induced angiogenesis, neovascularization and further restored blood flow in different ischemiamodels [27, 31, 32]. Supplement with exogenous kallikrein was also reported to enhance angiogenesis in the subventricular zone and peri-infarcted region in ischemic rats [21]. Herewe found that HUK increased capillary density in cerebral peri-infarct area (Fig 2C and 2D). In addition, using ^18^F-FDG PET/CT, our study showed that HUK enhanced cerebral perfusion in the penumbra region of experimental stroke rats (Fig 2A and 2B).

In generation of novel vessels, kininsgenerated from kallikren on kininogenhave been reported to initiate multiple signaling pathways through its interaction with B1 and B2 receptors. Accumulating evidence indicate that tissue kallikrein/kinin through kinin B2 receptor activation promotes angiogenesis through Akt-GSK-3β-VEGF-VEGFR-2 and Akt-eNOS-NO signaling pathways [11]. The present study provides novel insight into the mechanisms mediating HUK-induced angiogenesis. HUK potentially facilitated tube formation and branches of vessels through up-regulating ERK1/2-apelin-APJ-VEGF pathway.

Amplified VEGF and apelin/APJ were observed in stroke rat brain (Fig 3). In cultured endothelial cells, ERK pathway acted as a convergence point for inducing apelin/APJ and VEGF, for ERK selective inhibitor U0126 partially abolished HUK’s effects on apelin/APJ and VEGF expression (Fig 4A–4G). Increased ERK1/2 phosphorylation has been confirmed by our previous study in experimental ischemic mouse brain [13]. This study is in agreement with previous study showing a positive correlation between tissue kallikrein and ERK1/2 activation [13, 28, 33].

Although both of VEGF and apelin are established pro-angiogenic cytokines, the crosstalk between VEGF and apelin is complicated and has not been in consensus yet. In the retinal vascularization development,only minor differences were found in VEGF expression between apelin-KO and wild-type mice [34]. Previous evidence suggests that apelin manifests angiogenic properties independent of the VEGF signaling pathway [35], but also can act in cooperation with VEGF in inducing vessel sprouting and blood flow restoration [36]. In this study, VEGF expression was partially blocked by the apelin/APJ inhibitor in cultured endothelial cells, which indicatedthat VEGF expression was in part dependent on apelin/APJ activity (Fig 4H and 4I).

Taken together, our study has initially confirmed that HUKexertsprotective and angiogenic effects on rat stroke model. Furthermore, we here provide novel insight into mechanisms underlying HUK’s roles in post-stroke angiogenesis. That is. HUK-enhanced expression of VEGF and apelin/APJ in ERK1/2 dependent waymay serve as an alternative mechanism for inducing angiogenesis after cerebral ischemia.

## Supporting Information

S1 Fig HUK had no effect on the level of major physiological parameters in stroke rats. Major physiological parameters including blood gas, heart rate and blood pressure were monitored before, during and after MCAO surgery in HUK-treated MCAO rats. No significant differences were noticed in (A) Blood gas, (B) heart rate and (C) blood pressure, including systolic blood pressure (SBP), diastolic blood pressure (DBP) and mean blood pressure (MBP) in HUK-treated stroke rats. N = 10 per group.
(TIF)
