# Supplementary figures and images for: Human Urinary Kallidinogenase Promotes Angiogenesis and Cerebral Perfusion in Experimental Stroke
Source: PLoS One. 2015 Jul 29;10(7):e0134543. doi: 10.1371/journal.pone.0134543 (PMC4519127; doi:10.1371/journal.pone.0134543)

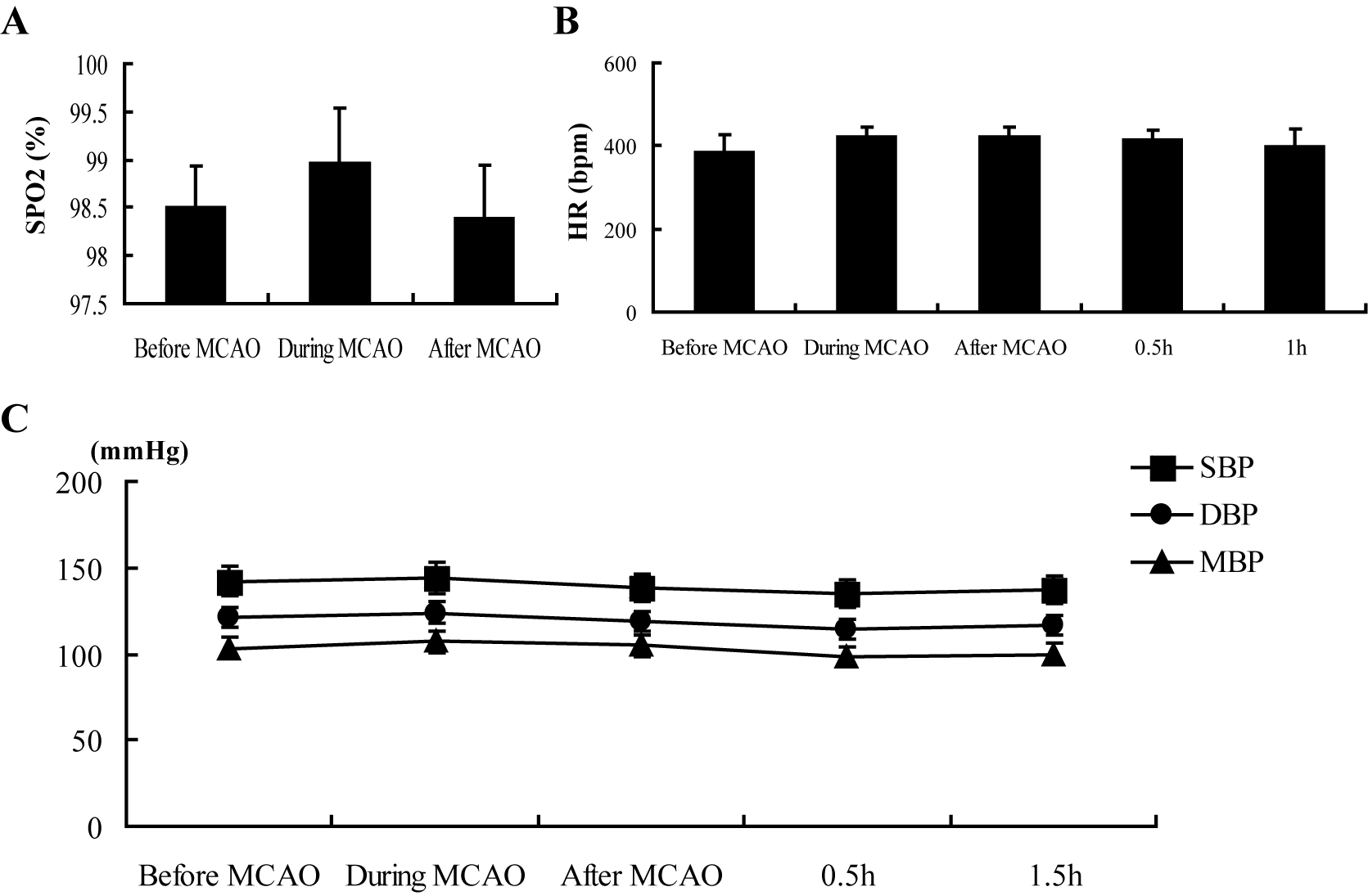

Supplement: S1 Fig — Major physiological parameters including blood gas, heart rate and blood pressure were monitored before, during and after MCAO surgery in HUK-treated MCAO rats. No significant differences were noticed in (A) Blood gas, (B) heart rate and (C) blood pressure, including systolic blood pressure (SBP), diastolic blood pressure (DBP) and mean blood pressure (MBP) in HUK-treated stroke rats. N = 10 per group. (TIF) [file pone.0134543.s001.tif]
